# Variations in Temperature Sensitivity (*Q*_10_) of CH_4_ Emission from a Subtropical Estuarine Marsh in Southeast China

**DOI:** 10.1371/journal.pone.0125227

**Published:** 2015-05-28

**Authors:** Chun Wang, Derrick Y. F. Lai, Chuan Tong, Weiqi Wang, Jiafang Huang, Chongsheng Zeng

**Affiliations:** 1 Key Laboratory of Humid Sub-tropical Eco-geographical Process of Ministry of Education, Fujian Normal University, Fuzhou, China; 2 School of Geographical Sciences, Fujian Normal University, Fuzhou, China; 3 Department of Geography and Resource Management, and Centre for Environmental Policy and Resource Management, The Chinese University of Hong Kong, Shatin, New Territories, Hong Kong SAR, China; 4 Institute of Geography, Fujian Normal University, Fuzhou, China; Fudan University, CHINA

## Abstract

Understanding the functional relationship between greenhouse gas fluxes and environmental variables is crucial for predicting the impacts of wetlands on future climate change in response to various perturbations. We examined the relationships between methane (CH_4_) emission and temperature in two marsh stands dominated by the *Phragmites australis* and *Cyperus malaccensis*, respectively, in a subtropical estuarine wetland in southeast China based on three years of measurement data (2007–2009). We found that the *Q*_10_ coefficient of CH_4_ emission to soil temperature (*Q*_s10_) from the two marsh stands varied slightly over the three years (*P* > 0.05), with a mean value of 3.38 ± 0.46 and 3.89 ± 0.41 for the *P*. *australis* and *C*. *malaccensis* stands, respectively. On the other hand, the three-year mean *Q*_a10_ values (*Q*_10_ coefficients of CH_4_ emission to air temperature) were 3.39 ± 0.59 and 4.68 ± 1.10 for the *P*. *australis* and *C*. *malaccensis* stands, respectively, with a significantly higher *Q*_a10_ value for the *C*. *malaccensis* stand in 2008 (*P* < 0.05). The seasonal variations of *Q*_10_ (*Q*_s10_ and *Q*_a10_) differed among years, with generally higher values in the cold months than those in the warm months in 2007 and 2009. We found that the *Q*_s10_ values of both stands were negatively correlated with soil conductivity, but did not obtain any conclusive results about the difference in *Q*_10_ of CH_4_ emission between the two tidal stages (before flooding and after ebbing). There were no significant differences in both *Q*_s10_ and *Q*_a10_ values of CH_4_ emission between the *P*. *australis* stand and the *C*. *malaccensis* stands (*P* > 0.05). Our results show that the *Q*_10_ values of CH_4_ emission in this estuarine marsh are highly variable across space and time. Given that the overall CH_4_ flux is governed by a suite of environmental factors, the *Q*_10_ values derived from field measurements should only be considered as a semi-empirical parameter for simulating CH_4_ emissions.

## Introduction

Methane is a greenhouse gas that is 34 times more potent than carbon dioxide on a 100-year time scale and hence plays an important role in global climate change [1]. Natural wetlands in particular are the single largest global CH_4_ source [2]. The global interannual variability of CH_4_ emissions are primarily driven by fluctuations of CH_4_ emissions from natural marshes, which on average emitted 177–284 Tg CH_4_ yr^-1^ during the period of 2000–2009 [1]. While numerous researchers have examined CH_4_ dynamics in northern peatlands [3–7], relatively little has been done in the coastal and estuarine wetland ecosystems [8–10]. It is essential to develop a thorough understanding of the relationships between various environmental factors and CH_4_ emissions from estuarine wetlands in order to accurately predict the impacts of natural and anthropogenic perturbations on CH_4_ release, as well develop appropriate management strategies to minimize the potential adverse climatic impacts of wetlands.

The net CH_4_ emission from wetland soil is a reflection of the balance between CH_4_ production, oxidation and transport. Temperature is in general a major factor governing wetland CH_4_ emission to the atmosphere [6, 11, 12], although some researchers have reported a weak correlation between CH_4_ emission and temperature [13, 14]. The *Q*_10_ coefficient has been commonly used to describe the temperature response of various microbial-mediated processes by standardizing temperature-related differences in reaction rates to proportional changes per 10°C rise in temperature. It is also considered to be one of the most important parameters used to assess the apparent temperature sensitivity of both soil respiration and ecosystem respiration [15–19]. However, few studies thus far have reported on the *Q*_10_ of wetland CH_4_ emission [6, 20, 21].

As soil respiration is widely regarded to be related exponentially to temperature, an exponential function is often used to determine the *Q*_10_ of soil and ecosystem respirations. A number of studies have shown that *Q*_10_ of soil respiration is not constant during the year, and that *Q*_10_ tends to decrease with increasing temperature in both forest [22, 23]and grassland ecosystems [24]. Yet, it is not known whether *Q*_10_ of CH_4_ emission from wetland ecosystems will exhibit a similar pattern. In estuarine wetlands, tidal flow is a unique and important physical process that can influence various biogeochemical processes. While CH_4_ emission from a tidal marsh was found to vary among different tidal stages [25], there is hitherto a lack of studies that compare *Q*_10_ of CH_4_ emission in tidal marshes among different tidal stages and different years. It is believed that a better understanding of *Q*_*1*0_ variations for both carbon dioxide and CH_4_ fluxes could significantly improve our knowledge on the carbon balance of coastal estuarine wetland ecosystems [26].

In this study, we measured CH_4_ fluxes continuously over a 3-year period from two stands dominated by *Phragmites australis* and *Cyperus malaccensis*, respectively, in a tidal marsh in southeast China to address the following objectives: (1) to examine the relationship between CH_4_ emission and temperature in the two marsh stands; (2) to determine the seasonal and interannual variability of *Q*_10_ of CH_4_ emission from this tidal marsh; (3) to investigate the influence of two tidal stages (before flooding and after ebbing) on the *Q*_10_ value of CH_4_ emission; and (4) to elucidate on the influence of different vegetation on the *Q*_10_ value of CH_4_ emission.

## Materials and Methods

**Fig 1:**
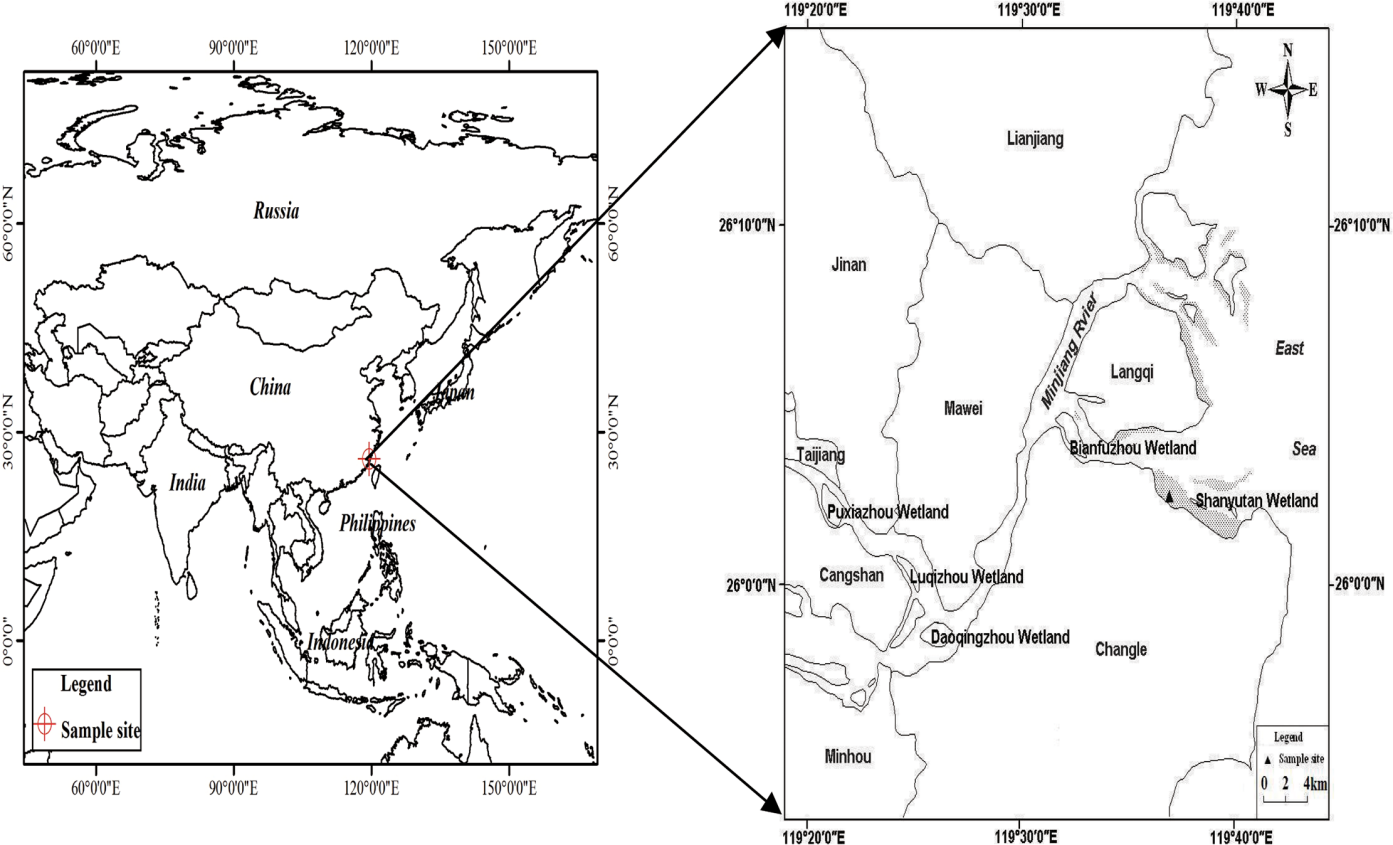
Location of the sampling sites.

The study was conducted in a tidal marsh at the Shanyutan wetland (26°00′36″–26°03′42″N, 119°34′12″–119°40′40″E, Fig 1) in the Min River estuary of Fujian Province in southeast China, with a total area 3120 ha (Fig 1). The climate of this subtropical region is warm and wet, with a mean annual temperature of 19.6°C and a mean annual precipitation of approximately 1,350 mm. Semi-diurnal tides are typical in the coastal area [27]. The soil surface is submerged for about 7 h over a 24 h cycle. There is normally between 10 and 150 cm of water above the soil surface at high tide. At other times the soil surface is completely exposed to air.

The study site was located in the midwest section of the Shanyutan wetland, with *C*. *malaccensis* and *P*.*australis* (Cav.) Trin as the dominant plant species. We randomly selected two monoculture marsh stands dominated by *C*. *malaccensis* and *P*.*australis*, respectively, with almost identical environmental conditions for flux measurements. The characteristics of the two stands are shown in Table 1. Vegetation and soil properties were determined in 2007. In three replicate quadrats (50 x 50 cm) of both the *P*. *australis* and *C*. *malaccensis* stands, the above-ground and belowground (0–60 cm depth) biomass were measured every two months, and every season, respectively. All biomass was oven-dried at 80°C to constant mass and weighed. Soil total organic carbon (TOC) content (0–50 cm depth) was measured via wet combustion of sediments in H_2_SO_4_/K_2_Cr_2_O_7_ [27,28].

**Table 1:** Vegetation and soil properties of the two marsh stands dominated by *P*. *australis* and C. malaccensis, respectively.

	*P*. *australis*	*C*. *malaccensis*
**Mean stem height (cm)**	149.9a	109.3b
**Mean plant density (individuals m** ^**-2**^ **)**	150a	912b
**Maximum above-ground live biomass (g m** ^**-2**^ **)**	1524.8 ± 78.9a	1062.4 ± 129.6b
**Annual mean below-ground biomass (0–60 cm) (g** ^**-2**^ **)**	2085.7 ± 663.0a	3168.4 ± 486.4b
**Soil organic carbon concentration (0–50 cm) (g kg** ^**-1**^ **)**	19.30± 1.17a	22.06±2.0a
**Soil organic carbon stock (0–50 cm) (kg m** ^**-2**^ **)**	7.76a	7.40a

Different letters represent a significant difference between the two stands.

The closed, static chamber technique [29] was used to measure CH_4_ emission from the two stands during two tidal stages in which the soil surface was exposed (i.e. before flooding, BF; after ebbing, AE). The maximum height of *C*. *malaccensis* was approximately 1.5 m, and the height of *P*. *australis* ranged from 1.6 to 1.8 m. The chambers consisted of three parts: a stainless steel bottom collar (50 cm length × 50 cm width x 30 cm height) and two individual PVC chambers (50 cm length × 50 cm width), with the lower and upper sections being 120 cm and 50 cm tall, respectively, for the *P*. *australis* stand, and 100 cm and 50 cm tall, respectively, for the *C*. *malaccensis* stand. The bottom collar was inserted permanently into the marsh sediment, with 2 cm left protruding above the sediment surface, while the PVC chambers were then placed on top of the collar during flux measurement. PVC chambers are commonly used in measuring wetland CH_4_ fluxes since they are opaque and can reduce overheating of the chamber headspace over the deployment period. However, a recent study suggested that the use of opaque chambers could lead to underestimation of CH4 fluxes from plants that transport gases actively through convection (e.g. *Phragmites* spp.) [30]. The top chamber was equipped with an electric fan to ensure a complete mixing of air inside the chamber headspace. Also, in the hot summer, the chamber top was covered by cotton quilts during flux measurements to keep the temperature inside within -0.8°C to 1.2°C of the ambient level. A wooden boardwalk was installed permanently throughout the three years of study to facilitate access to the measurement sites without causing significant disturbance during sampling.

Monthly CH_4_ flux measurements were made from January 2007 to December 2009, with the exception of February in these three years (owing to the Chinese Lunar New Year). Three replicate chambers separated by about 5 m were deployed in each marsh stand for gas sampling. All samples were taken on the days between the spring and neap tides (i.e. the third or fourth day after the largest spring tide). On these dates, the sampling sites began to flood at 10:00 am (Beijing time) and the soil was exposed to air again after ebb tide at about 1:30 pm. Chambers were deployed at 9:00 am (one hour before the beginning of flooding), and at approximately 3:00 pm (1.5 h after the end of the ebb tide) to determine the CH_4_ fluxes at two different tidal stages in a single day (before flooding, BF, and after ebbing, AE). To measure CH_4_ fluxes, three gas samples inside the chamber headspace were collected at 30-min intervals by 100 ml polypropylene syringes equipped with a three-way stopcock.

On each sampling date, one set of environmental variables was measured for each plant stand. Soil temperature, pH and redox potential at a depth of 10 cm were measured using a Eh/pH/temperature meter (IQ Scientific Instruments, USA), while soil conductivity (mS·cm^-1^) was measured using an electrical conductivity meter (2265FS, Spectrum Technologies Inc., USA). Air temperature (1.5 m above ground) was measured by a pocket weather meter (Kestrel-3500, USA).

Methane concentrations in the gas samples were determined using a gas chromatograph (Shimadzu GC-2010, Japan) equipped with a FID detector within 48 h after sampling. The column and detector temperatures were set at 60°C and 130°C, respectively, with nitrogen as the carrier gas at a flow rate of 20 ml min^-1^. The gas chromatograph was calibrated with gas standards containing 1.01, 7.99, and 50.5 μl CH_4_ l^-1^, respectively, on a monthly basis (i.e. every time when gas samples were analyzed). CH_4_ emission into the atmosphere was estimated by linear regression of the change in headspace CH_4_ gas concentrations with time [6]. The fluxes were rejected and removed from the analysis when the *R* ^*2*^ value of the linear regression was smaller than 0.90 [31].

We calculated the *Q*_10_ value of CH_4_ emission based on the exponential function that was commonly used to determine *Q*_10_ of soil and ecosystem respiration [24] as well as CH_4_ flux [6, 21], which was given as follows:

$$F=a{\text{e}}^{bt}$$ (1)

where *F* is CH_4_ efflux (mg m^-2^ h^-1^), *t* is the air temperature or soil temperature measured at 10 cm depth, and *a* and *b* are regression coefficients (*b* is also called the temperature reaction coefficient).

The *Q*_10_ value was then calculated as:

$$Q_{10}=e^{10b}$$ (2)

where *Q*_s10_ and *Q*_a10_, are the *Q*_10_ values based on soil and air temperatures, respectively.

The entire data set of each year was divided into two groups for analysis based on the timing of data collection, with one group in the warm months (warmer period between April to September) and the other in the cold months (colder period between January to March, and October to December). We determined the *Q*_10_ values of CH_4_ emission separately for these two groups of data. We also calculated the *Q*_10_ values of CH_4_ emission for the two different tidal stages.

All statistical analyses were performed using SPSS 16.0 software (SPSS Inc., Chicago, Illinois). The differences in vegetation, soil properties, and Q_10_ between the two marsh sites dominated by *P*. *australis* and *C*. *malaccensis* were tested using the paired-sample T test. The relationships between Q_s10_ values and other soil parameters were tested using the Pearson correlation analysis. We tested for any significance differences in *Q*_10_ among different years and seasons from the two stands using one-way ANOVA with Tukey’s post-hoc test.

## Results

### Relationship Between CH_4_ Flux and Temperature

**Fig 2:**
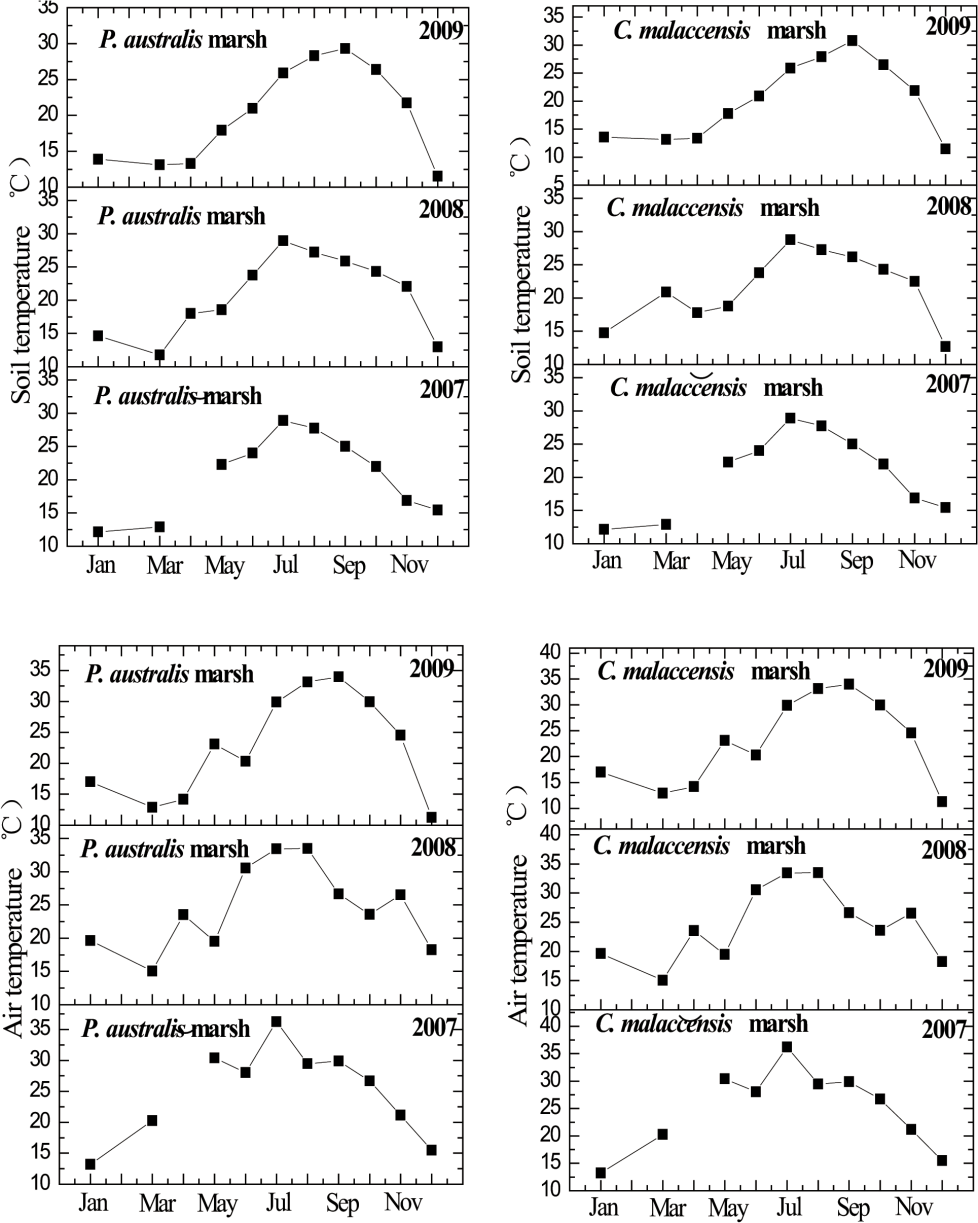
Monthly mean soil temperature (°C) at 10 cm depth or air temperature (°C) in the two marsh stands from 2007 to 2009. Data of soil (air) temperature was missing in April 2007 due to instrument failure.

**Fig 3:**
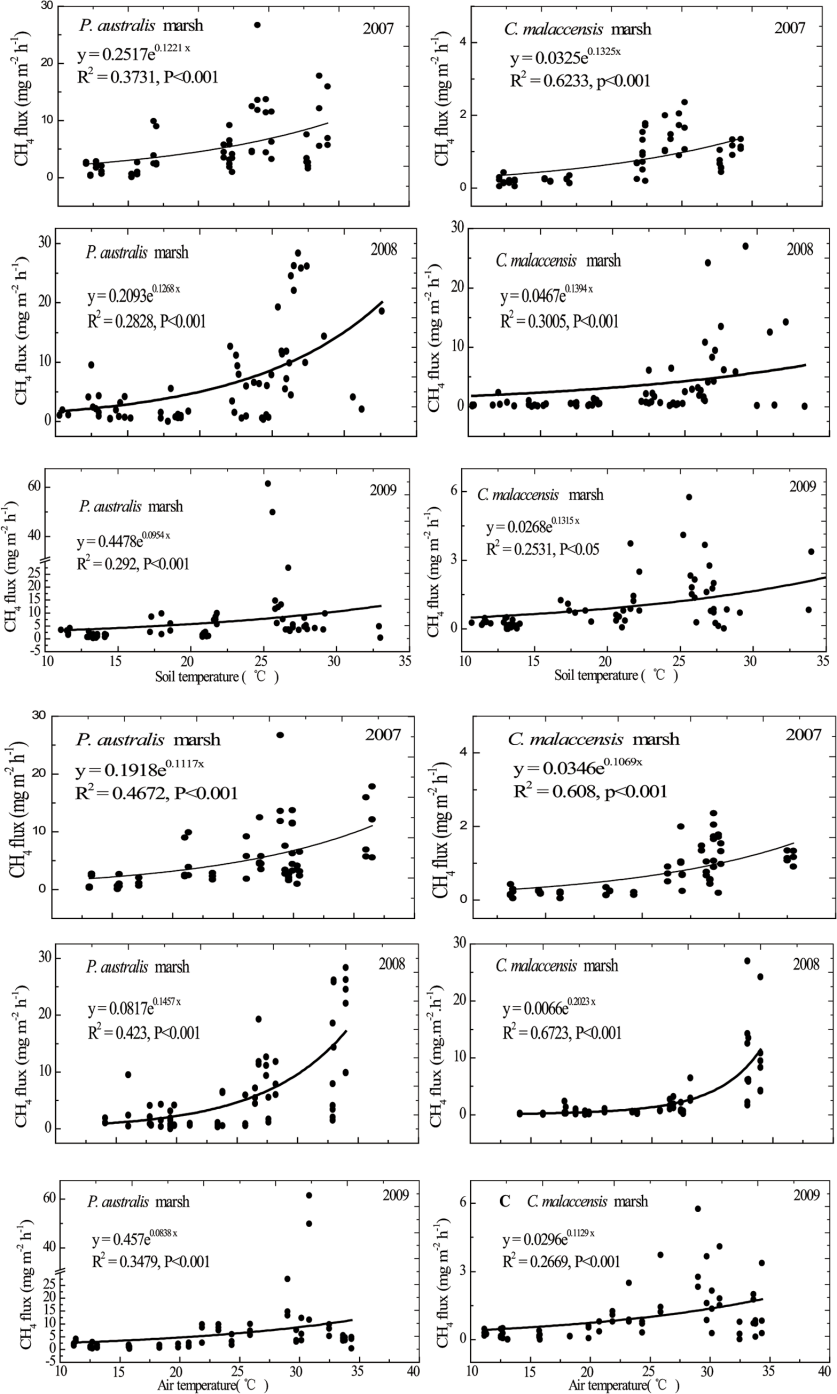
Relationships between CH_4_ emission (mg m^-2^ h^-1^) and soil temperature (°C) at 10 cm depth or air temperature (°C) in the two marsh stands from 2007 to 2009 as described by the exponential function (P < 0.05).

Variations of soil and air temperature from the *P*. *australis* and *C*. *malaccensis* marshes during 2007 to 2009 are shown in Fig 2. In the three years of our study, annual mean CH_4_ emissions from the *P*. *australis* stand ranged from 5.26 ± 0.67 to 6.41 ± 0.94 mg m^-2^ h^-1^, with minimum and maximum fluxes of 0.10 and 61.40 mg m^-2^ h^-1^, respectively. For the *C*. *malaccensis* stand, annual mean CH_4_ emissions ranged from 0.84 ± 0.12 to 2.97 ± 0.65 mg m^-2^ h^-1^, with minimum and maximum fluxes of 0.01 and 27 mg m^-2^ h^-1^, respectively. The temporal variation of CH_4_ fluxes from the *P*. *australis* stand and the *C*. *malaccensis* stand had been reported previously [27]. CH_4_ emission from the *P*. *australis* stand was significantly higher than that of the *C*. *malaccensis* stand (*P* < 0.05, Fig 3). The relationship of CH_4_ emissions with both soil temperatures at a depth of 10 cm and air temperature could be described significantly by the exponential function (*P* < 0.01, Fig 3). Except in 2009, the percentage variance in CH_4_ emission explained by air or soil temperature was greater for the *C*. *malaccensis* stand compared to the *P*. *australis* counterpart (Fig 3).

### Inter-annual Variations in Q_10_ Values

**Fig 4:**
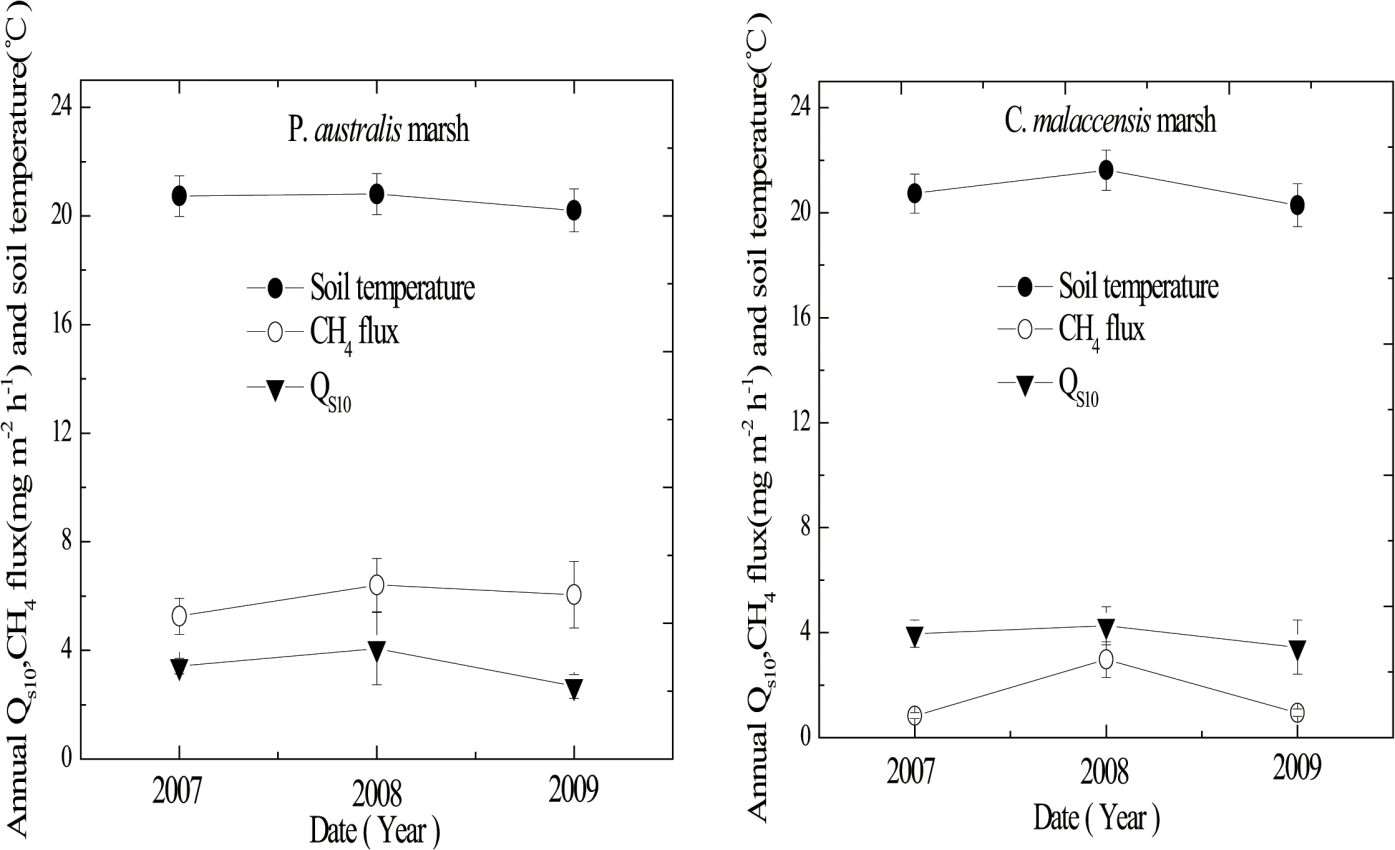
Interannual variations of *Q*_s10_ values (solid triangle), CH_4_ emission (open circle) and soil temperatures at 10 cm depth (solid circle) in the two marsh stands from 2007 to 2009. Error bars represent 1 standard error of the means.

**Fig 5:**
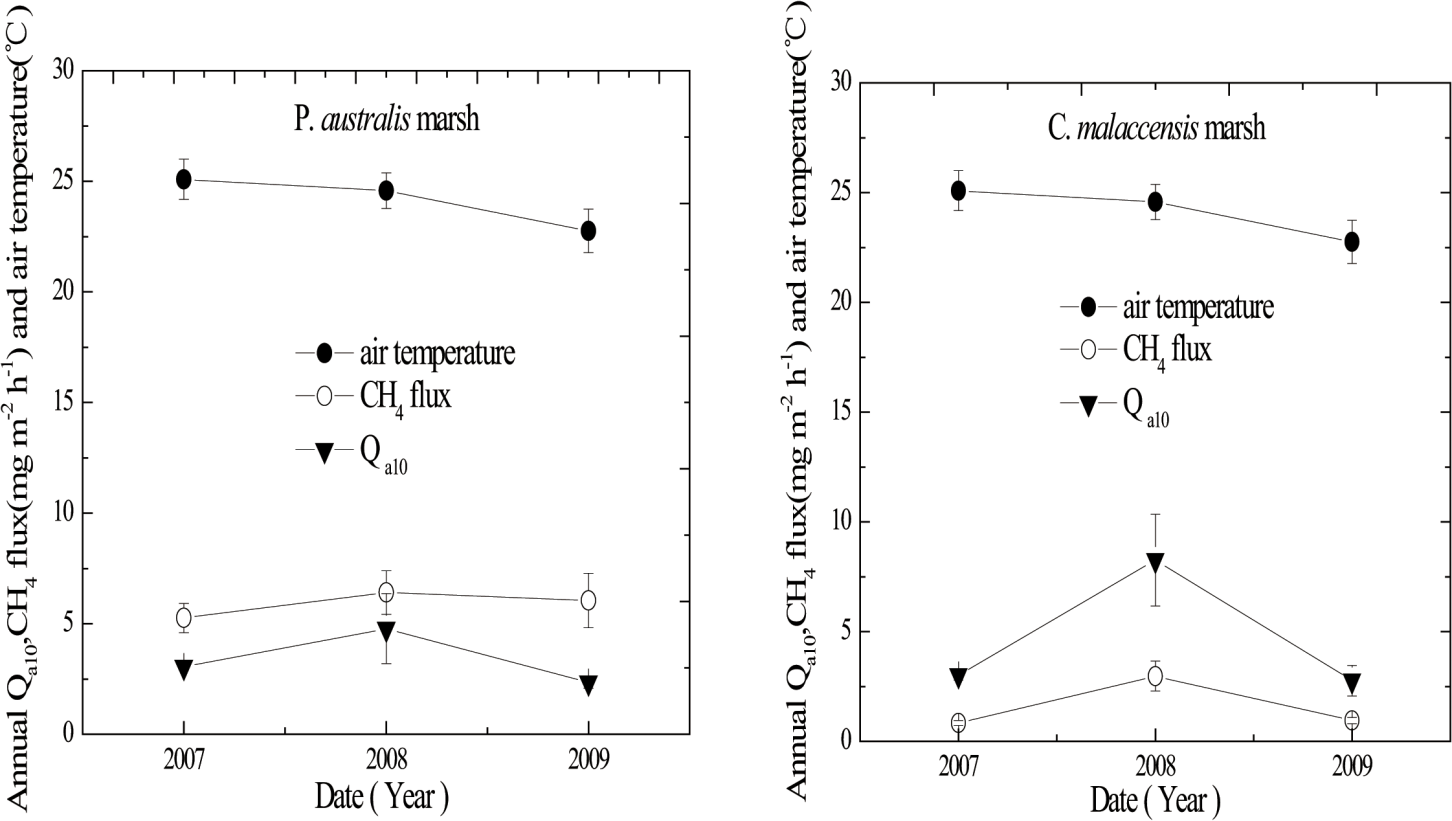
Interannual variations of *Q*_a10_ values (solid triangle), CH_4_ emission (open circle) and air temperatures (solid circle) in the two marsh stands from 2007 to 2009. Error bars represent 1 standard error of the means.

The *Q*_s10_ values of CH_4_ emission from the two marsh stands showed little inter-annual variations in the three study years (*P* > 0.05, Fig 4). Annual mean *Q*_s10_ values of CH_4_ emission from *P*. *australis* stand were 3.41 ± 0.29, 4.07 ± 1.33 and 2.67 ± 0.45 in 2007, 2008 and 2009, respectively, while those from *C*. *malaccensis* stand were 3.96 ± 0.53, 4.26 ± 0.73 and 3.45 ± 1.04, respectively. In general, the *Q*_s10_ values from the *P*. *australis* stand was lower than that of the *C*. *malaccensis* stand (*P* > 0.05). On the other hand, for *Q*_a10_ of CH_4_ emissions, the values from *C*. *malaccensis* stand were significantly higher in 2008 compared to the other two years (*P* < 0.05), while that from *P*. *australis* stand were not distinct different (*P* > 0.05). In 2007–2009, annual mean *Q*_a10_ of CH_4_ emission from *P*. *australis* stand was 3.06 ± 0.04, 4.78 ± 1.58 and 2.35 ± 0.27, respectively, while that from the *C*. *malaccensis* stand was 3.02 ± 0.24, 8.26 ± 2.09 and 2.76 ± 0.69, respectively. The annual mean CH_4_ fluxes from the two stands were also highest in 2008, with values of 6.41 ± 0.94 and 2.97 ± 0.65 mg m^-2^ h^-1^ for the *P*. *australis* and *C*. *malaccensis* stands, respectively. The variations in *Q*_s10_ and *Q*_a10_ of the two stands among these three years were consistent with CH_4_ emission, except for the *Q*_s10_ of the *P*. *australis* stand (Figs 4 and 5).

### Seasonal Variations in Q_10_ Values

**Fig 6:**
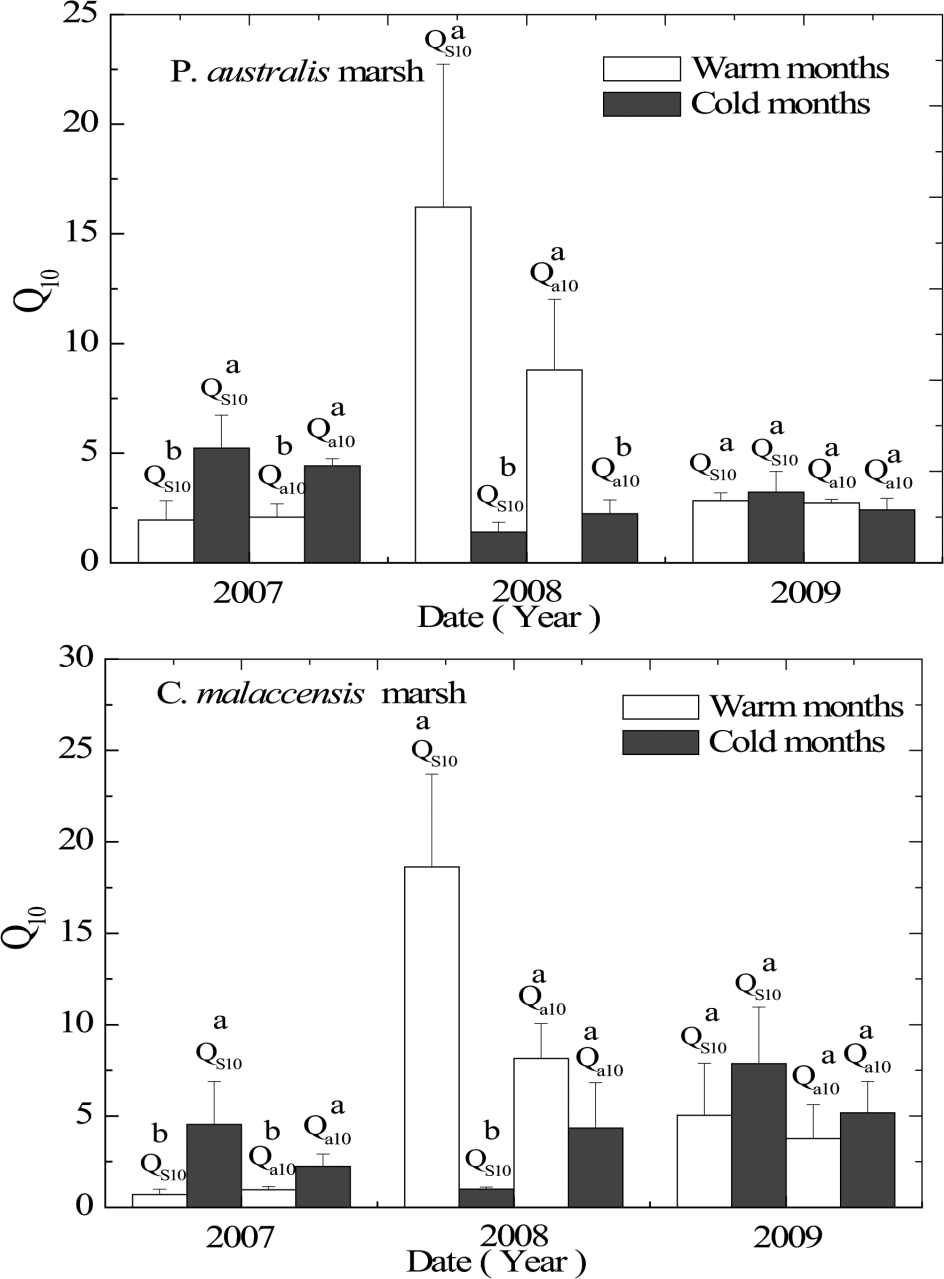
*Q*_s10_ and *Q*_a10_ of CH_4_ emissions from the two marsh stands in the warm months and cold months from 2007 to 2009. Values are represented by means of triplicates ± 1 standard error. Significant differences in *Q*_s10_ or *Q*_a10_ (P < 0.05) between the two periods are indicated by different letters.

For the two marsh stands, both *Q*_s10_ and *Q*_a10_ generally exhibited a distinct difference between the warm and cold months except in 2009 (Fig 6). In 2007, both *Q*_s10_ and *Q*_a10_ of CH_4_ emission from the two marsh stands were considerably lower in the warm months compared to those in the cold months (*P* < 0.05, Fig 6), particularly for the *Q*_a10_ of CH_4_ emission from the *P*. *australis* stand (*P* < 0.05, Fig 6). In 2009, both *Q*_s10_ and *Q*_a10_ from the *C*. *malaccensis* stand were also slightly lower in the warm months (*P* > 0.05), but the seasonal difference was less discernible for the *P*. *australis* stand (*P* > 0.05, Fig 6). In contrast, the *Q*_s10_ values of CH_4_ emission from the two stands in 2008 were significantly higher in the warm months than in the cold months (*P* < 0.05).

### Difference in Q_10_ Values Between Two Tidal Stages

**Fig 7:**
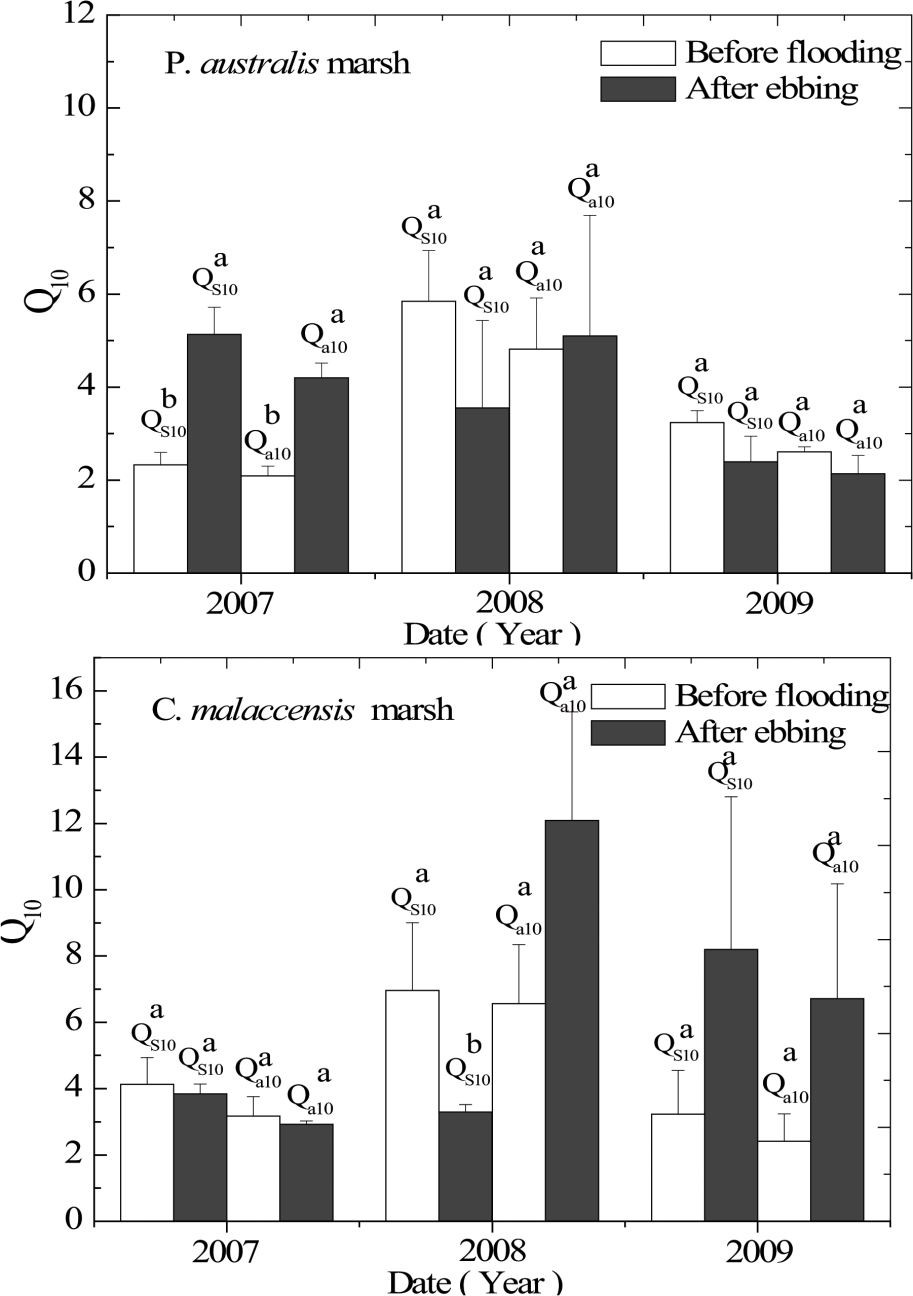
*Q*_s10_ and *Q*_a10_ of CH_4_ emission from the two marsh stands in two tidal stages (before flooding and after ebbing) from 2007 to 2009. Values are represented by means of triplicates ± 1 standard error. Significant differences in *Q*_s10_ or *Q*_a10_ (P < 0.05) between the two tidal stages are indicated by different letters.

Fig 7 shows the mean *Q*_s10_ and *Q*_a10_ of CH_4_ emission from the two marsh stands in two tidal stages (before flooding and after ebbing) over three years. In 2007, we found significantly lower *Q*_s10_ and *Q*_a10_ of CH_4_ emission from the *P*. *australis* stand before flooding (BF) compared to those after ebbing (AE) (*P* < 0.05), while for the *C*. *malaccensis* stand, the difference was not statistically significant between the two tidal stages (*P* > 0.05). In 2008, the *Q*_s10_ and *Q*_a10_ values of both stands were higher during BF and AE, respectively, yet the difference was only statistically significant for the *Q*_s10_ value in the *C*. *malaccensis* stand (*P* < 0.05). In 2009, no significant difference in both *Q*_s10_ and *Q*_a10_ values were observed between the two tidal stages in the two stands (*P* > 0.05), although we observed considerably higher mean values during AE compared to BF in the *C*. *malaccensis* stand.

### Relationships between Q_s10_ values and other soil parameters

The *Q*_s10_ values were not significantly different among the three years of study in both marsh stands (*P* > 0.05). Therefore, the *Q*_s10_ values in the three different years trial could be treated as replicates of the stands. Correlation analysis shows that the *Q*_s10_ of both stands were negatively correlated with soil conductivity, but positively correlated with soil redox potential (Table 2). The *Q*_s10_ of CH_4_ emission from the *P*. *australis* stand was positively correlated with soil pH, while an opposite relationship was observed in the *C*. *malaccensis* stand (Table 2).

**Table 2:** Pearson correlation coefficients between Q_s10_ of CH_4_ emissions and soil properties from the two marsh stands.

Properties	*P*. *australis*		*C*. *malaccensis*	
	*r*	*p*	*r*	*p*
**Conductivity**	-0.204	*p*>0.05	-0.141	*p*>0.05
**pH**	0.321	*p*>0.05	-0.641	*p*>0.05
**Redox**	0.015	*p*>0.05	0.483	*p*>0.05

## Discussion

### Relationship Between CH_4_ Flux and Temperature

In our study, the annual mean CH_4_ emission ranged from 5.26 ± 0.67 to 6.41 ± 0.94 mg m^-2^ h^-1^ for the *P*. *australis* stand, and 0.84 ± 0.12 to 2.97 ± 0.65 mg m^-2^ h^-1^ for the *C*. *malaccensis* stand, which was consistent with previous findings of a lower CH_4_ emission associated with a higher salinity condition [32]. This was likely a result of the high availability of electron acceptors (e.g. sulfate, nitrate) in seawater that completely eliminated methanogens and shifted the dominant anaerobic pathways of organic carbon mineralization [33,34]. Moreover, we found that CH_4_ emission rate in our tidal wetland study site was lower than that in a tidal freshwater marsh from the Daoqingzhou wetland upstream of the Minjiang estuary, China, but within the range reported in the estuarine wetlands in the Yangtze River in China and Sundarban in India [35,36] (Table 3). The exponential model expressed the relationships between CH_4_ emission and temperature fairly well (Fig 3), which was consistent with findings in a boreal forest wetland in Saskatchewan Canada [37] and a high *P*. *australis* marsh of the Wuliangsu Lake, Inner Mongolia, northern China [38]. Temperature governs soil biogeochemical processes directly by altering microbial metabolism which is largely driven by enzymatic kinetics, or indirectly by controlling substrate availability [39–41]. Numerous studies [37, 42–44] have demonstrated a positive relationship between CH_4_ emission and temperature as a result of the increased C availability at higher temperatures. Meanwhile, the influence of temperature on CH_4_ emission is very complex as the overall CH_4_ emission from wetland soils to the atmosphere is a net result of CH_4_ production, oxidation, and transport, which could be independently controlled by temperature or other factors such as precipitation, soil carbon input amount and quality, soil texture, etc. [40,41].

**Table 3:** Summary of methane emission rates from estuarine wetlands reported in previous studies.

Sites	Dominated vegetation types	Salinity(ppt) /Conductivity (mS cm^-1^)	CH_4_ emission (mg m^-2^ h^-1^)	References
Daoqingzhou wetland, Minjiang estuary, China	*Cyperus malaccensis*	0.31~0.50 (mS cm^-1^)	14.75± 2.32	(unpublished data)
Chongxi wetland, Yangtze River estuary, China	*Phragmites australis*	<0.5 for most time, MAX<1.5(ppt)	1.322~11.660	[35]
Sundarban mangrove estuaries, Bay of Bengal, India	mangrove	0.3~ 3.1(ppt)	0.001~ 12.240	[36]
Shanyutan wetland, Minjiang estuary, China	*C*. *malaccensis* and *P*.*australis* (Cav.) Trin	3.11~4.22 (mS cm^-1^)	0.84~6.41	This study
The northern Yellow River estuary, China	*S*. *salsa*	22~31(ppt)	-0.035~ 0.055	[32]

### Inter-annual Variations in Q_10_ Values

Understanding the response of soil biogeochemical processes to a warmer world is critical for predicting short-term and long-term changes in the cycling of soil carbon and nitrogen. Field measurements of both the magnitude and temperature sensitivity of CH_4_ emission are important for understanding methane dynamics in wetlands. We observed the highest *Q*_10_ values of CH_4_ emission from the tidal marsh stands dominated by *P*. *australis* and *C*. *malaccensis* in 2008, with the *Q*_s10_ values of 4.07 ± 1.33 for the *P*. *australis* stand and 4.26 ± 0.73 for the *C*. *malaccensis* stand, and the *Q*_a10_ values of 4.78 ± 1.58 for the *P*. *australis* stand and 8.26 ± 2.09 for the *C*. *malaccensis* stand, respectively (Figs 4 and 5). The higher temperature sensitivity of CH_4_ emission from both stands in 2008 was likely the result of a higher soil temperature in 2008 than the other two years (Fig 4), which could speed up the dissolution and diffusion of substrates [41,42]. At the same time, the annual mean CH_4_ fluxes from the two stands were found to be highest in 2008. Interannual variability of *Q*_*10*_ was not significant among the three years in both stands (*P* > 0.05), except for *Q*_a10_ in the *C*. *malaccensis* stand with significantly higher value in 2008. The high temporal and spatial heterogeneity of soil metabolism might have partly masked some possible differences in mean *Q*_s10_ values of the two stands among years [40]. Updegraff *et al*. [45] found a large variation in *Q*_10_ of CH_4_ production potential among the soils of northern wetlands, ranging from *Q*_10_ values of 1.98 in sedge meadow to 16.2–28.0 in various peat soils to a depth of 1 m, which implied a strong temperature-substrate interaction influencing methanogenic metabolism. In a review of the potential rate of CH_4_ production, Segers [46] reported a high variability of *Q*_10_ for methanogenesis in wetland soils, with an overall mean of 4.1 ± 0.4 and a range of 2–28 and 1.5–6.4 for minerotrophic and oligotrophic peat soils, respectively. A very wide range of *Q*_10_ of CH_4_ production between 1 and 35 has been observed in some acid mire soils [47]. Valentine *et al*. [48] found that the higher *Q*_10_ coefficients (1.7 to 4.7) observed for methanogenesis in peat slurries were related to the lower lignin to nitrogen ratios.

### Seasonal Variations in Q_10_ Values

We found that the *Q*_10_ values (both of the *Q*_s10_ and *Q*_a10_ values) of CH_4_ emission for the *P*. *australis* stand and the *C*. *malaccensis* stand were higher in the cold months than those in the warm months during 2007 and 2009, which was consistent with the findings of previous studies [16;22–24;49], with the exception of *Q*_a10_ values from the *P*. *australis* stand. The *Q*_10_ value reflects the apparent temperature sensitivity of the underlying microbial processes involved in CH_4_ production. Temperature has been found to govern microbial populations more strongly at lower temperatures [16, 49]. Temperature affects both production and turnover of extracellular enzymes in soils [50], and thus possibly indirectly alters the *Q*_s10_ values of CH_4_ emission. The *Q*_10_ value of *Methanosarcina barkeri*, which was able to metabolize both acetate and H_2_-CO_2_ in the production of CH_4_, was found to range from 1.3 to 4 [51], while methanogenic bacteria in rice paddy soils had a *Q*_10_ value of up to 12 [52]. The large variation of CH_4_ production could be due to the anomalous temperature behavior of the methanogens themselves as well as the interactions between several distinct microbial processes [46]. Dunfield *et al*. [53] reported a higher *Q*_10_ value for CH_4_ production (5.3–16) compared to that of CH_4_ oxidation (1.4–2.1) in some northern peat soils which suggested that the *Q*_10_ of the overall CH_4_ emission may be further affected by the different temperature responses of methanogens and methanotrophs due to different enzymatic processes. Several studies also have indicated that the temperature sensitivity of extracellular enzymes varied seasonally [54,55]. In this study, the seasonal pattern of *Q*_10_ values (both *Q*_s10_ and *Q*_a10_) of CH_4_ emission for the two stands in 2008 was opposite to that observed in 2007 and 2009 (Fig 6). Unfortunately, we cannot provide a credible explanation for this variability because we did not have any information regarding the influence of different temperature ranges on methanogen populations to provide further insights regarding the causes for the seasonal variations in *Q*_10_ values of CH_4_ emission during the three years.

### Difference in Q_10_ Values Between Two Tidal Stages

We did not obtain any conclusive results whether there was a consistent difference in the *Q*_10_ of CH_4_ emission between the two tidal stages (BF and AE) (Fig 7). A considerable temporal variation in CH_4_ emission was found, with a higher flux during BF in some months but a greater emission during AE in some other months. In a 4-year study, Chang and Yang [8] also showed that the monthly CH_4_ emissions were sometimes higher during BF, whereas in other months, they were higher during AE. This inherently high temporal variability of CH_4_ emission during BF and AE would further add to the difficulty of examining the influence of tidal stages on the temperature sensitivity of CH_4_ emission in the two marsh stands. Salinity is an important stressor factor in coastal marshes through its effects primarily on ionic strength and the microbial pathway of soil carbon mineralization [33]. Neubauer [34] found that the *Q*_10_ of CH_4_ emission were comparatively lower in the added salt plots (*Q*_10_ = 1.6–2.5) than that in the added fresh plots (*Q*_10_ = 3.2–3.5) at Brookgreen Gardens tidal freshwater marsh, USA. In our study, the *Q*_s10_ values of both stands were negatively correlated with soil conductivity, although statistically not significant (*P* > 0.05) (Table 2). The annual mean soil conductivity was 3.58–4.29 mS cm^-1^, with no significant difference between BF and AE (*P* > 0.05). Soil pH is another major variable that could govern the ionization of organic molecules, as well as the activity and function of enzymes [56]. Min et al. [56] found that the C-acquiring β-glucosidase (βGase) activity was higher in more alkaline conditions regardless of soil temperature, but the temperature sensitivity of βGase was higher at pH 4.5. In our study, we found lower soil pH values in the two stands during AE than at BF, although the range of annual mean was small (6.44–6.78) and some of the differences were not significant statistically. Soil redox potential could also profoundly affect CH_4_ production and emission from wetland soils as methanogens are anaerobic microorganisms, but we found no significant correlation between soil redox potential and the *Q*_s10_ of CH_4_ emission in both stands during BF and AE (*P* > 0.05). Overall, our results suggest that the temporal and spatial variations of soil properties may exert little influence on the *Q*_10_ of CH_4_ emission over the short term between the two tidal stages.

### Difference in Q_10_ Values Between Two Marsh Stands

It is known that some wetland plants capable of convective transport substantially influence CH_4_ emission by providing a pathway for gases through aerenchyma [57]. Simultaneously, aerenchyma tissues of wetland plants could transport oxygen to the anaerobic root zone [30]. In our study, we found the CH_4_ fluxes from the *P*. *australis* stand were higher than those from the *C*. *malaccensis* stand, which could be attributed to a better developed aerenchyma system in *P*. *australis*. However, it is hard to extrapolate this effect of wetland vegetation to temperature sensitivity (*Q*_10_ values) of CH_4_ emission owing to the complicated biogeochemical processes. It is possible that CH_4_ emission induced by convective transport in wetland plants is susceptible to changes in the local micro-environment (e.g. air temperature and relative humidity) [58]. Alternatively, wetland plants physiological functions (such as transpiration, photosynthesis and respiration) may also contribute to the CH_4_ metabolic processes [30]. Song *et al*. [6] found significant difference in *Q*_10_ values of CH_4_ emission between the *Carex lasiocarpa* and *Calamagrostis angustifolia* marshes of the Sanjing Plain, northeastern China (2.50 vs. 1.90). In our study, we also found lower mean *Q*_10_ values (*Q*_s10_ and *Q*_a10_) of CH_4_ emission for the *P*. *australis* stand when compared with the *C*. *malaccensis* stand, albeit the difference was not significant. No significant differences in the annual *Q*_10_ values (*Q*_s10_ and *Q*_a10_) of CH_4_ emission were found among the three study years for both stands (*P* > 0.05), with the exception of *Q*_a10_ for the *C*. *malaccensis* stand (*P* < 0.05) (Figs 4 and 5). Meanwhile, the *Q*_s10_ values of CH_4_ emission determined in our tidal wetlands were found to be higher than those in the freshwater marshes in the Sanjiang Plain of northeast China (2.67–4.26 vs. 2.49), which suggests a stronger positive climatic feedback to warming in the subtropical brackish marshes compared to the freshwater counterparts in the temperate region. On the other hand, the *Q*_s10_ values in the peat bogs of Moorehouse Nature Reserve in North Pennines, UK as well as the paddy fields in Hangzhou and Taoyuan of China were higher than those in our estuarine marshes (5.2–5.93, Table 4), which might be related to differences in root exudation, methanotrophic communities, etc. that deserve further investigation.

**Table 4:** Summary of Q_s10_ values of methane emissions from different types of wetlands reported in previous studies.

Sites	Wetland types	Q_s10_ values	References
Wuliangsu,Inner Mongolia,China	Lake wetlands	3.17~5.07	[59]
Sanjiang Plain, Northeast China	Freshwater marshes	2.49	[60]
Hangzhou,Zhejiang and Taoyuan, Hunan,China	Rice fields	5.93	[61]
Moorhouse Nature Reserve, North Pennines,UK	Peat bogs	5.2	[62]
The Shanyutan wetland, Minjiang Estuary, China	Tidal brackish water marshes	2.67~4.26	This study

## Conclusions

In summary, we found that CH_4_ emission from the two tidal marsh stands in the Min River estuary increased exponentially with both soil and air temperatures. Both *Q*_s10_ and *Q*_a10_ exhibited a strong seasonal pattern, yet the variations were not consistent among different years. We also observed differences in *Q*_10_ of CH_4_ emission between the two tidal stages, with the pattern being quite variable from one year to the other. Meanwhile, we found a lower *Q*_10_ values (*Q*_s10_ and *Q*_a10_) of CH_4_ emission for the *P*. *australis* stand compared with the *C*. *malaccensis* stand, although the difference was not statistically significant.

Although measurements in the field has the advantage of being more realistic than laboratory assays, our results suggest that the *Q*_10_ values of CH_4_ emission derived from field data should generally be regarded as a semi-empirical fitting parameter for simple models only as the fluxes determined reflect a combination of a number of processes (e.g. substrate production, methane production, oxidation and transmission). *Q*_10_ is actually only an indicator of the apparent temperature sensitivity, with the actual fluxes in the field being affected by a suite of factors like temperature, root biomass quantity and activity, moisture conditions, and perhaps other unknown variables. In view of the large variability of the temperature response of CH_4_ emissions over space and time, a longer-term monitoring with more frequent measurements might be necessary to obtain a better understanding of the variations of *Q*_10_ values. Given the paucity of data on *Q*_10_ of CH_4_ emission from the subtropical region, our findings provided some useful data on the temperature sensitivity to better predict the response of CH_4_ emission to future climate change.
